# Ruthenium(II)-Arene Curcuminoid Complexes as Photosensitizer Agents for Antineoplastic and Antimicrobial Photodynamic Therapy: In Vitro and In Vivo Insights

**DOI:** 10.3390/molecules28227537

**Published:** 2023-11-11

**Authors:** Emanuela Marras, Camilla J. Balacchi, Viviana Orlandi, Enrico Caruso, Maurizio F. Brivio, Fabrizio Bolognese, Maristella Mastore, Miryam C. Malacarne, Miriam Rossi, Francesco Caruso, Veronica Vivona, Nicole Ferrario, Marzia B. Gariboldi

**Affiliations:** 1 Department of Biotechnology and Life Sciences (DBSV), University of Insubria, Via JH Dunant 3, 21100 Varese, Italy; emanuela.marras@uninsubria.it (E.M.); camillajoelle@gmail.com (C.J.B.); viviana.orlandi@uninsubria.it (V.O.); enrico.caruso@uninsubria.it (E.C.); fabrizio.bolognese@uninsubria.it (F.B.); mc.malacarne1@uninsubria.it (M.C.M.); vvivona@studenti.uninsubria.it (V.V.); nferrario1@studenti.uninsubria.it (N.F.); 2 Department of Theoretical and Applied Sciences (DiSTA), University of Insubria, Via JH Dunant 3, 21100 Varese, Italy; maurizio.brivio@uninsubria.it (M.F.B.); maristella.mastore@uninsubria.it (M.M.); 3 Department of Chemistry, Vassar College, Poughkeepsie, NY 12604, USA; rossi@vassar.edu (M.R.);

**Keywords:** PDT, aPDT, ruthenium(II)-arene curcuminoids, colon cancer cell lines, *Galleria mellonella*

## Abstract

Photodynamic therapy (PDT) is an anticancer/antibacterial strategy in which photosensitizers (PSs), light, and molecular oxygen generate reactive oxygen species and induce cell death. PDT presents greater selectivity towards tumor cells than conventional chemotherapy; however, PSs have limitations that have prompted the search for new molecules featuring more favorable chemical–physical characteristics. Curcumin and its derivatives have been used in PDT. However, low water solubility, rapid metabolism, interference with other drugs, and low stability limit curcumin use. Chemical modifications have been proposed to improve curcumin activity, and metal-based PSs, especially ruthenium(II) complexes, have attracted considerable attention. This study aimed to characterize six Ru(II)-arene curcuminoids for anticancer and/or antibacterial PDT. The hydrophilicity, photodegradation rates, and singlet oxygen generation of the compounds were evaluated. The photodynamic effects on human colorectal cancer cell lines were also assessed, along with the ability of the compounds to induce ROS production, apoptotic, necrotic, and/or autophagic cell death. Overall, our encouraging results indicate that the Ru(II)-arene curcuminoid derivatives are worthy of further investigation and could represent an interesting option for cancer PDT. Additionally, the lack of significant in vivo toxicity on the larvae of *Galleria mellonella* is an important finding. Finally, the photoantimicrobial activity of **HCurc I** against Gram-positive bacteria is indeed promising.

## 1. Introduction

Photodynamic therapy (PDT) is a promising medical technique for the treatment of certain types of tumors and localized bacterial infections. PDT uses photosensitizers (PSs), a light source, and molecular oxygen to produce reactive oxygen species (ROS), which exert cytotoxic action on cancer cells and bacteria. Minimal normal tissue toxicity, reduced systemic effects, and a lack of intrinsic or acquired resistance mechanisms are some of the advantages that PDT has over classical chemotherapy. However, the clinical applications of PDT are still limited, and this strategy does not apply to the treatment of metastasized cancer and large hypoxic tumors [1]. The ideal PS should have certain characteristics, such as very low toxicity in the absence of light (dark), optimal absorption in the 600–900 nm window, no production of toxic metabolites, accumulation, and targeting of cell organelles, a low rate of photobleaching, optimal absorption, distribution, and excretion properties.

Three interrelated mechanisms are involved in the antitumor effects of PDT: direct killing of tumor cells, vasculature damage induction, and stimulation of the immune system. The relative contribution of these mechanisms is related to the type and dose of PS used, the time between PS administration and photoactivation, the total light dose and its fluence rate, the oxygen concentration, as well as the nature of the tumor cells. PDT can also be used in combination with chemotherapy and radiotherapy without any adverse effects [2]. PSs based on a tetrapyrrolic molecular scaffold are the most used clinically. However, these compounds have some limitations, such as high lipophilicity, photobleaching, low body clearance, and low selectivity.

Recent advancements have underscored the potential of PDT combined with antibiotics and/or disinfectants in an antibacterial antimicrobial-PDT (aPDT) strategy. In particular, it has been proposed as a strategy to combine with antibiotics and/or disinfectants for application in different fields, such as clinical, veterinary, nutritional, and environmental. Interestingly, aPDT inactivates microorganisms regardless of their antimicrobial resistance pattern and, due to its multitarget mechanism of action, manifests a low probability of selecting drug-resistant strains.

Interest in the development of new natural compounds to be used for anticancer and/or antibacterial treatment and PDT has exponentially increased. It is estimated that from 1981 to 2019, almost 25% of all newly registered anticancer drugs were obtained from natural products [3,4,5], and a 2019 review reports that about 400 compounds have been identified as possible candidates for use as PSs [6]. In this context, it has been shown that the biological effects of curcumin (**CUR**), isolated from *Curcuma longa* rhizomes, may be amplified through the combination with light [7], and different curcuminoids used in PDT significantly inhibit cell viability in cancer cell lines [8,9,10,11]. Furthermore, the photodynamic application of **CUR** and curcuminoid derivatives has been extensively investigated in microbial growth inhibition [12,13,14,15]. However, studies on the antitumoral PDT activity of **CUR** are far less advanced, likely because the absorption peak in the blue region (350–500 nm) of the molecule might limit the clinical applicability of **CUR** as a PS to superficial tumors only. Nevertheless, some PDT-**CUR** studies investigated the activity of tumor cells in vitro and in vivo. For example, one interesting result reported that the lipophilic nature of **CUR** promotes the incorporation of the drug within mammalian cells, causing significant PDT activity [16].

In general, **CUR** demonstrated PDT activity against oral, liver, skin, colon, kidney, prostate, bladder, breast, and cervical cancer through inhibition of tumor growth and activation of apoptotic pathways. Nonetheless, the range of tumors that can be treated with PDT-**CUR** is limited by its low water solubility, rapid metabolism, interference with other drugs, and low stability [16,17].

Efforts to optimize PSs for use in anticancer activity include the use of metal complexes [18]. Furthermore, advancements in nanotechnology currently offer interesting opportunities for novel therapeutic approaches. In particular, metal organic frameworks (MOFs) are multifunctional nanomaterials that are finding applications in PDT [19,20].

Platinum is the dominant element in the antineoplastic area, but ruthenium (Ru) is also promising due to the comparable ligand-exchange kinetics of Pt(II) and Ru(II) complexes in aqueous solutions. Moreover, ruthenium can mimic iron binding to biomolecules, lowering toxicity and enhancing selectivity for cancer cells. Lastly, the entering clinical trials of two Ru(II) inorganic complexes (NAMI-A and KP1019) motivated further research in this direction [21,22]. Furthermore, metal-based PSs, especially Ru(II) complexes, have attracted substantial attention in PDT due to their particularly favorable chemical–physical characteristics, such as their low amount of photobleaching. In addition, the heavy atom effect originating through metal coordination ensures a greater tendency to form the excited triplet state and, thus, to produce increased quantities of ^1^O_2_. Interestingly, the Ru(II)-PS TLD-1433 is currently in phase II clinical trials for bladder cancer [23,24].

Other promising studies highlighting the biomedical features of Ru(II) complexes include the ability of Ru(II)-PSs to exert antibacterial effects against Gram-positive (i.e., MRSA biofilms) and Gram-negative (*E. coli*) bacteria [25]; the antineoplastic properties of half-sandwich (η6-arene)-Ru(II) complexes and Ru(II)-arene dimers with assorted ligands [26]; and antibiotic and antiviral activities for water-soluble forms of Ru(II)-arene complexes [27].

No attempts have been conducted on the use of these molecules as photosensitizers, however, and only limited endeavors have been made in conjugating the metal center with ligands having intrinsic biological activity. The aim of this work was a chemical and biological characterization of some Ru(II)-arene curcuminoids useful in anticancer and antibacterial PDT. Chemical analyses to evaluate the hydrophilicity, photodegradation rates, and singlet oxygen generation of the candidate drugs as PSs were performed. The photodynamic effects of the compounds were evaluated on human colorectal cancer cell lines, along with their ability to induce ROS production and apoptotic, necrotic, and/or autophagic cell death. In addition, the possible toxic effects of the Ru(II)-arene curcuminoids were investigated in vivo using the larvae of *Galleria mellonella* (Lepidoptera, Pyralidae, Gm) as an alternative to mammals [28] by evaluating the mortality rate of larvae over time.

The larvae of Gm have established themselves in both research and industrial fields, as studies on these invertebrates have provided results comparable to those obtained with mammals [29]. In particular, Gm appears to be a very promising model to investigate the antimicrobial efficacy and toxicity of various drugs [30,31]. Recently, Allegra et al. reported evidence that larvae can be a good model for assessing the toxicity of drugs, showing that the Lepidoptera provided a reliable LD_50_ assessment of acute chemical toxicity [32]. Furthermore, Gm larvae have several advantages, such as low cost, easy maintenance, and manipulation, as well as not being subjected to legal and ethical restrictions that hinder the use of vertebrates (Directive n. 2010/63/UE; https://eur-lex.europa.eu/eli/dir/2010/63/oj, accessed on 2 January 2023; D.L. 26/2014; https://www.gazzettaufficiale.it/eli/id/2014/03/14/14G00036/sg, accessed on 2 January 2023). Moreover, our group has recently used Gm in a PDT study [33].

Last, for this work, the potential antimicrobial activity of **CUR** derivatives and their Ru(II)-arene complexes was tested in *Bacillus subtilis* ATCC 6633 and *Escherichia coli* MG1655, chosen, respectively, as Gram-positive and Gram-negative model microorganisms.

Overall, the results shown indicate that the Ru(II)-arene derivatives could represent an interesting alternative for anticancer PDT and that some of them could be used in aPDT.

## 2. Results and Discussion

### 2.1. Chemistry

**Figure 1:**
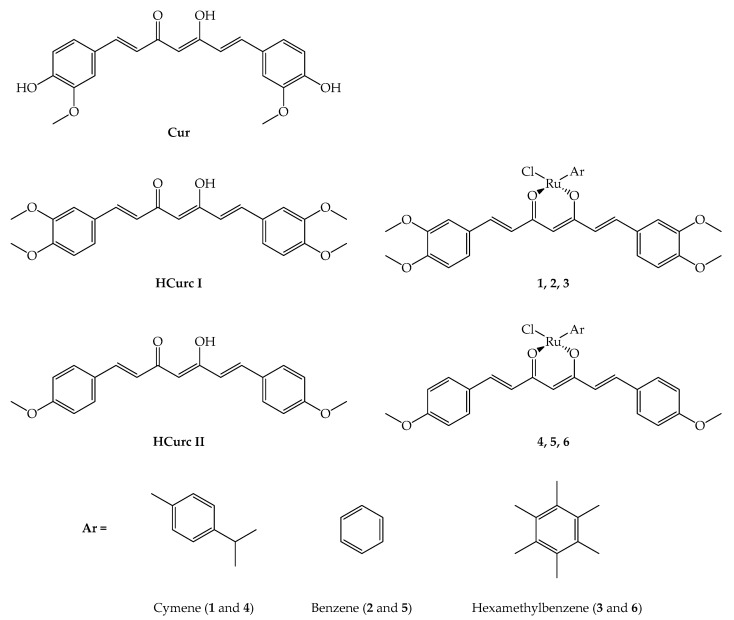
**CUR**, curcuminoids (**HCurc I** and **HCurc II**) and their Ru(II)-arene derivatives [34].

The synthesis of curcuminoid molecules (**HCurc I** and **HCurc II**) and of their Ru(II)-arene derivatives (**1**, **2**, **3**, **4**, **5**, and **6**; Figure 1) was previously reported [34].

Preliminary HPLC chromatographic studies were performed on **CUR**, curcuminoids, and their Ru(II)-arene derivatives to evaluate their stability (1 mM in DMSO). The results showed that **CUR**, **HCurc I**, and **HCurc II** are stable over time, while the Ru(II)-arene complexes are less stable. In particular, the complete degradation of the two complexes with cymene (**1** and **4**) was observed in two months. Given these results, all the chemical and biological analyses were carried out using fresh samples.

### 2.2. Photodynamic Characteristics and Stability

The photodynamic features of **CUR**; **HCurc I**, **1**, **2**, and **3**; and **HCurc II**, **4**, **5**, and **6**, in terms of lipophilicity and ^1^O_2_ generation capability, were characterized, along with the stability of all compounds.

The molecule partition coefficient (P) between 1-octanol and water is usually used to assess the lipophilicity of organic compounds. The values obtained from the UV-Vis analysis of both the organic and aqueous phases are generally expressed as LogP, and values greater than 3 indicate highly lipophilic molecules. However, with highly lipophilic (or, conversely, hydrophilic) compounds, this method cannot be applied, since the absorbance in one of the two phases is too low to be detected spectrophotometrically. Therefore, when it is not possible to apply spectroscopic methods, a lipophilicity scale can be obtained for chemical compounds belonging to a homogeneous series by measuring the HPLC retention time (Rt) and extrapolating the LogP values from calibration curves obtained with molecules with known LogPs. Specifically, p-anisaldehyde, anthracene, 9-anthracenylaldehyde, and benzaldehyde were used for this set of experiments. The LogP values shown in Table 1 were thus obtained from the standards’ chromatograms and the related calibration curves.

The two curcuminoids showed greater lipophilicity than **CUR** due to the lack of two OH groups; furthermore, **HCurc I** is less lipophilic than **HCurc II** because of the two additional methoxy groups [34]. In addition, when **HCurc I** and **HCurc II** were complexed with Ru(II)-arene, a decrease in lipophilicity was observed for all derivatives. Specifically, according to the different lipophilicity of the three arene groups coordinated with Ru(II) (hexamethylbenzene > cymene > benzene), derivatives **3** and **6** were the most lipophilic, and derivatives **2** and **5** were the least lipophilic of the series, while cymene-Ru(II) derivatives showed intermediate lipophilicity.

Singlet oxygen quantum yield values for **CUR**; **HCurc I**, **1**, **2**, and **3**; and **HCurc II**, **4**, **5,** and **6** were obtained indirectly by evaluating their decay kinetics using 1,3-Diphenylisobenzofuran (DPBF), a well-known singlet oxygen quencher, and using the equation reported in Material and Methods, in which **CUR** was used as a reference compound. It is well established that **CUR** is not a higher producer of singlet oxygen, being a quencher of this free radical [35], and that its ^1^O_2_ quantum yield value is 0.19 [36]. As shown in Table 1, none of the molecules tested produced higher singlet oxygen levels than **CUR**.

It is well known that highly stable PSs may induce higher and prolonged in vivo photodynamic activity compared to PSs that are rapidly decomposed through irradiation. These latter types of PSs can hardly be applied in clinical therapy, as their effectiveness may wear off before the complete treatment of the diseased tissue. The stability of the two curcuminoids and their Ru(II)-arene derivatives was assessed by irradiating a 100 µM aqueous solution of each compound for up to 2 h and evaluating their photodegradation. The results obtained were compared with those obtained for **CUR** and are reported in Table 2. In agreement with data reported by other authors [37,38], **CUR** has a high stability to irradiation with white light. Despite the fact that **HCurc I** and **HCurc II** were significantly less stable than **CUR**, showing complete molecule degradation in 30 min, their Ru(II)-arene derivatives (**1**–**6**) demonstrated higher stability, comparable to that of **CUR**. No other peaks appeared in the UV-Vis spectrum following the photoinduced degradation.

The behavior of **HCurc I** and **HCurc II** is in agreement with previous studies [36,39] and could be explained considering their molecular structure. Indeed, phenolic hydroxyl groups are free radical quenchers, which protect the molecule from the ROS formed during the irradiation process through their antioxidant activity [40]. Removing these groups from the two curcuminoids could result in the destabilization of the compounds’ diketone group and other double bonds that might become highly susceptible to breaking [41]. On the other hand, the greater stability observed for derivatives **1**–**6** could probably be attributable to the coordination of the metal with the enol group of the curcuminoid derivatives, leading to a less photodegradable molecule [42].

The test was also performed using a blue LED light for irradiation, with similar results. Considering the results obtained, to ensure a similar degree of photostability for all compounds, the irradiation step in biological tests was limited to 30 min.

### 2.3. Studies on Cancer Cells

#### 2.3.1. Effects on Cell Viability

Preliminary experiments showed no differences in the effect of the studied compounds on cell viability following photoactivation with both blue and white light (Supplementary Material Table S1). Furthermore, as reported in Table 3, the two curcuminoid molecules derived from **CUR**, namely **HCurc I** and **HCurc II**, showed similar or even better photodynamic performances compared to **CUR**. Based on these observations, all further investigations were performed using a halogen white lamp for the irradiation step, and **CUR** was no longer considered.

**Figure 2:**
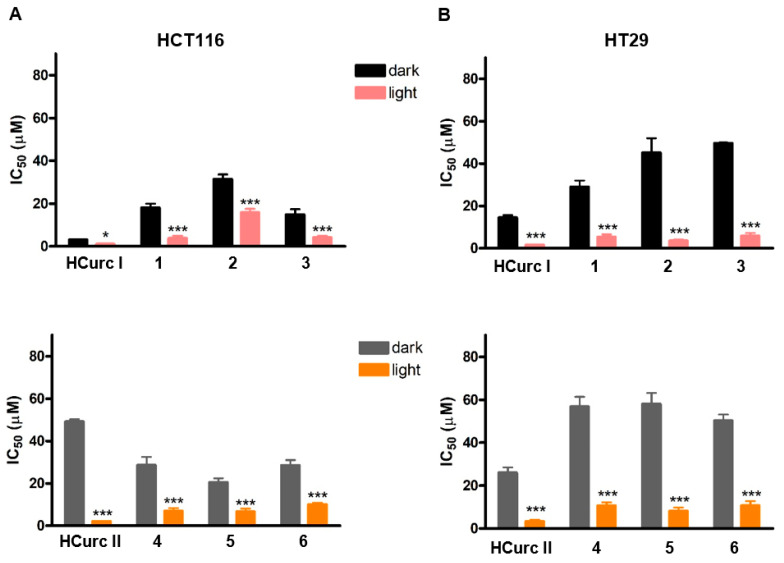
IC_50_ values obtained in HCT116 (**A**) and HT29 (**B**) cell lines following 24 h treatment with the studied compounds, 30 min irradiation under a white halogen lamp, 24 h incubation in drug-free medium, and MTT assay (mean ± ES 3/4 independent experiments; * *p* < 0.05 and *** *p* < 0.001 vs. dark).

The IC_50_ values obtained in HCT116 and HT29 cells following exposure to **HCurc I**, **HCurc II,** and their Ru(II)-arene derivatives for 24 h, 30 min irradiation, 24 h incubation in drug-free medium, and MTT assay are reported in Figure 2 and Supplementary Materials Figure S1. The intrinsic effect of the compounds was assessed by omitting the irradiation step from the treatment protocol and reported as “dark” in the figure.

Our earlier study demonstrated that all the tested compounds possessed intrinsic cytotoxic effects, and metal complexes derived from **HCurc I** exhibited stronger antitumor activity than those of **HCurc II** [34]. Despite the fact that the schedule of treatment in the present study is different from the previous one, this behavior is confirmed for HCT116 cells and observed also in the HT29 cells, as shown in Table 3, indicating that photoactivation is not an absolute requisite for the activity of these molecules. Moreover, all the Ru(II) derivatives of **HCurc I** are more potent than the **HCurc II** derivatives. Therefore, after replacing the **CUR** hydroxyl groups with methoxy groups, the obtained compound **HCurc I** and its Ru(II)-complexes have increased antitumor activity compared to **HCurc II** and its related complexes. However, when the compounds were irradiated with white light, a significant photodynamic effect was observed for all of them. Specifically, all the tested compounds increased their potency following photoactivation compared to the effects observed in the dark, as indicated by the significant reduction of the IC_50_ values.

Interestingly, the dark IC_50_ values of the tested compounds were on average higher in HT29 cells than in HCT116 cells, indicating a greater resistance of the former. Conversely, following PDT, both cell lines responded similarly to the treatment, resulting in a higher phototoxic index of the tested compounds on HT29 cells compared to HCT116 cells (Table 4). Interestingly, the cellular response to PDT was in no way related to the different arene substituents present in the compounds.

Several factors are known to influence the cellular response and the mode of cell death following PDT, including the type of PS, its cellular localization, and the light dose. Moreover, as observed for other types of anticancer treatments, the cellular response to PDT might also be influenced by the tumor suppressor protein p53 [43,44,45]. Nevertheless, controversial results about the dependence of cellular response on p53 in PDT have been reported, some indicating p53-dependent PDT-induced cell death and others demonstrating an increase in cell resistance in the absence of functional p53 [44,45,46,47,48]. Considering that HT29 and HCT116 cell lines harbor a different form of p53, a mutant in the former and a wild type in the latter, our results seem to indicate that their response to PDT could be independent of p53. However, the inconsistency in the published results has often been related to the use of cancer cell lines, which probably carry other genetic differences than p53 status [45,49]. Thus, to better clarify this last issue, we performed an MTT assay on the p53-null cell line HCT116 E6.

**Figure 3:**
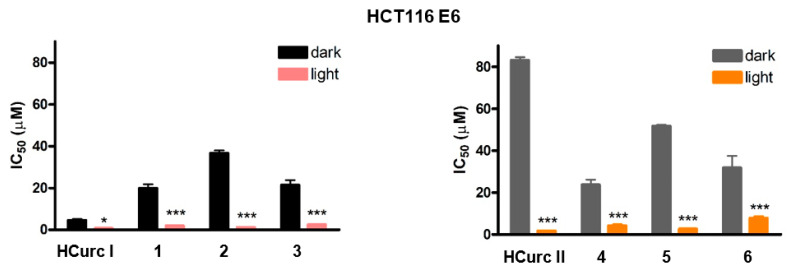
IC_50_ values obtained in HCT116 E6 cells following 24 h treatment with the studied compounds, 30 min irradiation under a white halogen lamp, 24 h incubation in drug-free medium and MTT assay (mean ± ES 3/4 independent experiments; * *p* < 0.05 and *** *p* < 0.001 vs. dark).

As shown in Figure 3, a strong photodynamic effect was observed following treatment with the studied compounds and PDT of the HCT116 E6 cells, with IC_50_ values similar to those observed for HCT116 and HT29 cells, confirming the irrelevance of p53 for the phototoxic effect. In addition, as reported in Supplementary Material Table S2, phototoxic indexes obtained for the Ru(II)-arene derivatives in the p53-null cell line were higher than those obtained for the p53 wild-type cell line.

#### 2.3.2. ROS Production

It is generally acknowledged that ROS generation is crucial to the successful application of PDT. Cellular response to PDT is associated with the type of photochemical reaction induced by the PS, which depends on the PS’s photophysical/photochemical nature, its subcellular localization, and the oxygen concentration [50].

Intracellular ROS production was evaluated using 2,7-dichlorodihydrofluorescein diacetate (DCFH-DA) as a probe, following treatment with concentrations of the studied compounds corresponding to their IC_50_ values for 24 h and 30 min irradiation.

**Figure 4:**
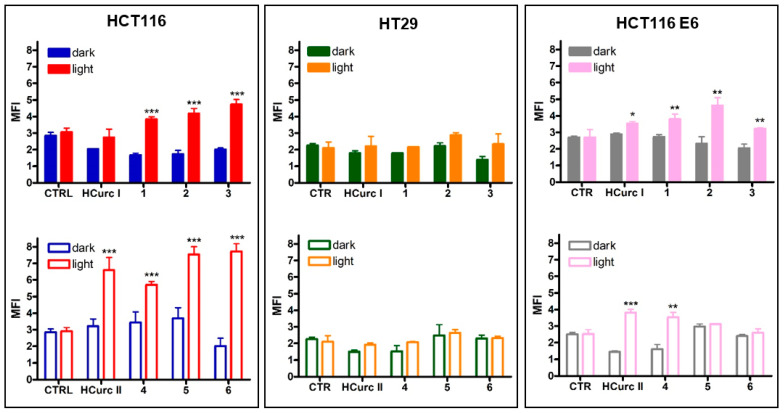
Intracellular ROS production following 24 h treatment with **HCurc I**, **HCurc II** and their Ru(II)-arene derivatives at concentrations corresponding to the respective IC_50_ values, 30 min irradiation, and 24 h incubation in drug-free medium (mean ± S.E. of 3 independent experiments; * *p* < 0.05, ** *p* < 0.01, and *** *p* < 0.001 vs. dark).

In the absence of irradiation, none of the compounds were able to induce an increase in ROS levels; however, significantly increased ROS levels were observed in **1**, **2**, **3**, **HCurc II**, **4**, **5**, and **6**-treated HCT116 cells following photoactivation. Similar behavior was also observed in HCT116 E6 cells. In contrast, HT29 cells appeared to be unable to produce ROS following treatment with the curcuminoids and their Ru(II) derivatives, both in the dark and upon irradiation (Figure 4). Thus, as reported for other PSs [51,52], the highest potency observed in HCT116 and HCT116 E6 cells following PDT compared to dark-maintained cells might be at least in part explained considering the ability of the compounds to increase ROS production upon irradiation.

#### 2.3.3. Cell Death Induction

**Figure 5:**
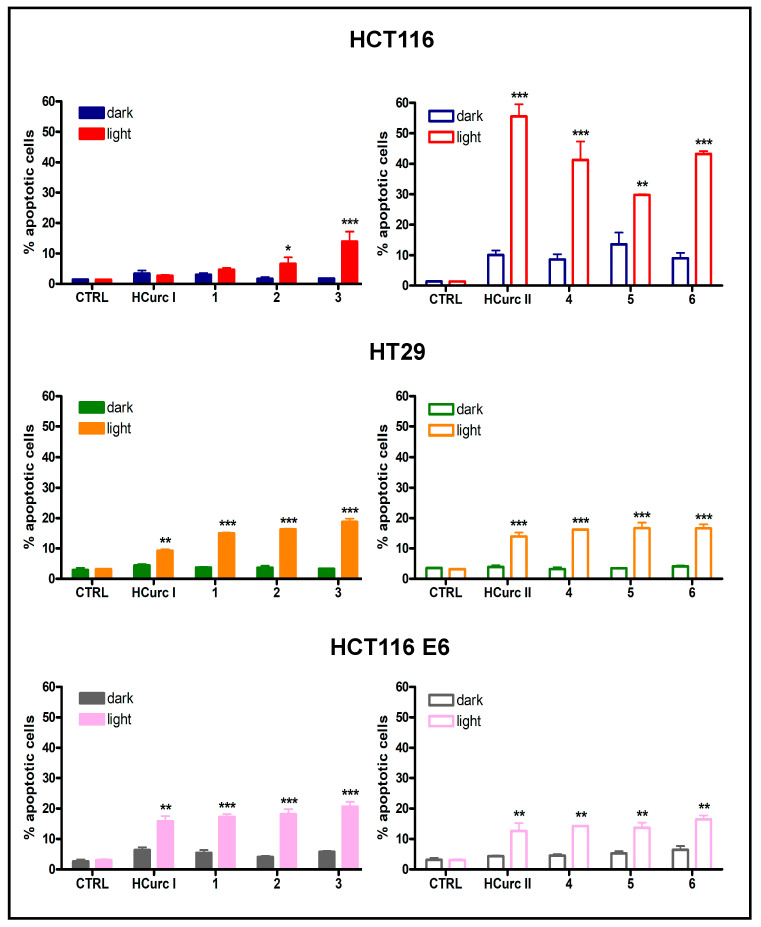
Percentage of apoptotic HCT116, HT29, and HCT116 E6 cells following 24 h treatment with **HCurc I**, **HCurc II,** and their Ru(II)-arene derivatives at concentrations corresponding to their IC_50_ values, 30 min irradiation, and 24 h incubation in drug-free medium (mean ± S.E. of 3 independent experiments; * *p* < 0.05, ** *p* < 0.01, and *** *p* < 0.001 vs. dark).

**Figure 6:**
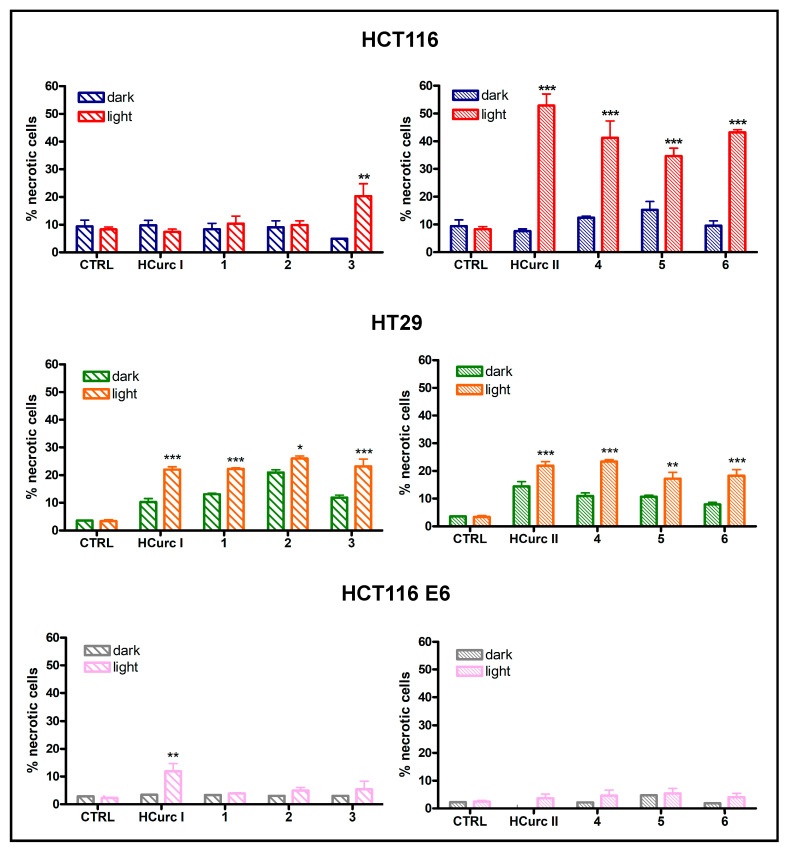
Percentage of necrotic HCT116, HT29, and HCT116 E6 cells following 24 h treatment with **HCurc I**, **HCurc II,** and their Ru(II)-arene derivatives at concentrations corresponding to their IC_50_ values, 30 min irradiation, and 24 h incubation in drug-free medium (mean ± S.E. of 3 independent experiments; * *p* < 0.05, ** *p* < 0.01, and *** *p* < 0.001 vs. dark).

It has been reported that PDT-induced killing of tumor cells involves different cell death mechanisms, such as apoptosis, necrosis, and ferroptosis [53]. In the absence of photoactivation, no significant increase in the percentages of apoptotic and necrotic cells was observed in all cell lines following treatment with equitoxic concentrations of the curcuminoids and their Ru(II)-arene derivatives. However, a significant increase in apoptotic and necrotic cell deaths over controls was induced following irradiation. Interestingly, as reported in Figure 5 and Figure 6, in HCT116 cells, the increase in levels of apoptotic and necrotic cells was more significant in cells treated with **HCurc II** and its Ru(II)-arene derivatives compared to the corresponding **HCurc I** derivatives, while in HT29 and HCT116 E6 cell lines, similar levels of apoptosis and necrosis were observed for both **HCurcI** and **HCurc II** Ru(II)-arene derivatives.

The generally low levels of apoptosis and necrosis induced by equitoxic concentrations of the compounds suggest that these two types of cell death might not be the only ones involved in the effects of the tested compounds and that their involvement might vary in a cell-specific way.

Recent experimental evidence indicates that PDT may induce autophagy-associated cell death [54,55,56], but the relationship between autophagy and cell death in PDT is still under discussion. As a matter of fact, it has been shown that an increase in autophagy upon PDT may lead either to cytoprotection or cytotoxicity in a cell-dependent manner [57,58]. Other authors have identified this pathway as responsible for the cell damage generated by different photosensitizers [56] and have shown that autophagic cell death only occurs upon the prevention of classical apoptosis [59].

**Figure 7:**
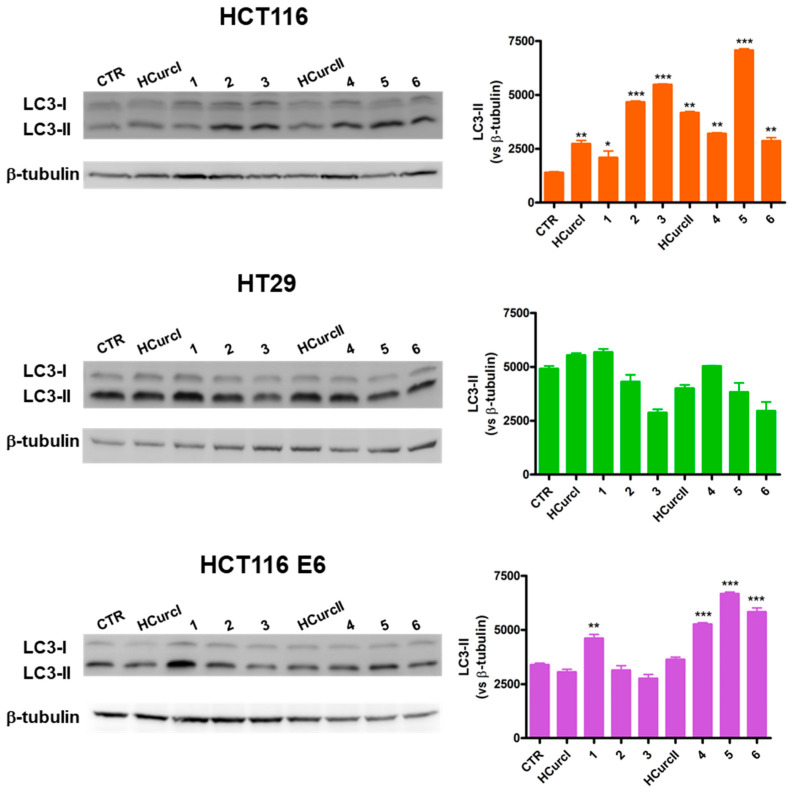
LC3-II protein levels (representative Western blot analysis out of 2 independent experiments with similar results) in the HCT116, HT29, and HCT116 E6 cell lines treated with **HCurc I**, **HCurc II,** and their Ru(II)-arene derivatives at concentrations corresponding to the IC_50_ values, 30 min irradiation, and 24 h incubation in drug-free medium, and relative densitometric analysis performed on all Western blot experiments (* *p* < 0.05; ** *p* < 0.01, and *** *p* < 0.001 vs. CTR).

LC3-II protein levels were thus assessed to address the possible ability of the two curcuminoids and their Ru(II)-arene derivatives to induce autophagy. The increase in LC3-II levels, which is a recognized autophagosomal marker, can indicate increased autophagy activation [60]. In Figure 7, two immunoreactive bands can be observed for LC3: the former corresponding to LC3-I (16 kDa), and the latter corresponding to LC3-II (14 kDa), resulting from the conjugation of LC3-I with phosphatidylethanolamine.

An increase in LC3-II was observed in HCT116 cells following treatment with all the studied compounds and PDT. Thus, in this cell line, **HCurc II** and its Ru(II) derivatives can induce either apoptosis, necrosis, or autophagy when photoactivated. Differently from what was observed in HCT116 cells, autophagy was not activated in HT29 in response to the treatment with the studied compounds. Furthermore, only **1**, **4**, **5,** and **6** seemed to be able to induce autophagy in HCT116 E6 cells.

Several studies have demonstrated that p53 modulates autophagy, although the relationship between them is still not well clarified. Recently, experimental observations reported that p53 can act both as an autophagy inhibitor and activator, depending on its cellular localization as well as its mode of action [61,62]. In general, our results do not seem to indicate a role of p53 in the autophagic response of HCT116, HT29, and HCT116 E6 cell lines to treatment with **HCurc I**, **HCurc II,** and their Ru(II)-arene derivatives.

### 2.4. In Vivo Toxicity on G. mellonella Larvae

Possible in vivo toxic effects of the studied derivatives were assessed by recording the mortality rate of *G. mellonella* viability up to seven days following intrahemocelic injection with the compounds. As negative controls, larvae were also injected only with a DMSO solution in PBS, or with sterile PBS, exposed to light or kept in the dark, and no toxic effects were observed.

**Figure 8:**
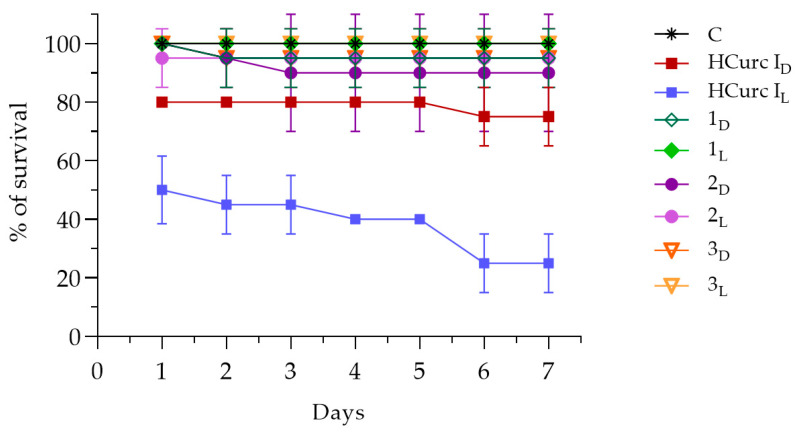
Survival of Gm larvae (%) following administration of **HCurc I**, **1**, **2,** and **3** in the dark or after PDT (mean ± S.D. of 4 independent experiments; C: control, i.e., PBS injection; D subscript: dark condition; L subscript: after PDT).

Figure 8 and Supplementary Material Table S3 show the effects of **HCurc I** and its Ru(II)-arene derivatives, namely **1**, **2,** and **3**, both after PDT (L) and in the dark (D), up to seven days after treatment. The data showed that, in the dark, **HCurc I** treatment resulted in Gm larvae survival of 75%, while following PDT further toxicity was evidenced, resulting in an overall survival of 25%. Interestingly, treatment with **1**, **2,** and **3** exerted only low toxic effects, both in the dark and after PDT, resulting in 90% survival.

**Figure 9:**
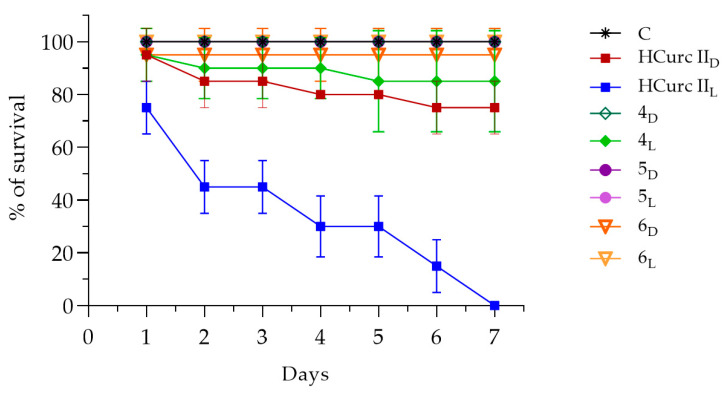
Survival of Gm larvae (%) following administration of **HCurc II**, **4**, **5,** and **6** in the dark or after PDT (mean ± S.D. of 4 independent experiments; C: control, i.e., PBS injection; D subscript: dark condition; L subscript: after PDT).

Even following treatment with **4**, **5,** and **6,** the observed toxicity was lower than that of the progenitor molecule (**HCurc II**), as the survival of the larvae always exceeded 85%, both in the dark and following PDT. On the other hand, only 75% of Gm larvae survived up to 7 days following **HCurc II** treatment in the dark, while total mortality of the larvae was observed following PDT (Figure 9 and Supplementary Material Table S4).

Studies about the toxicity of **CUR** and **CUR** derivatives were already performed on alternative models, such as *G. mellonella*, showing no toxicity in vivo in these animal models [63,64,65]. Our results are partially in agreement with those of these authors. Differently from them, we observed high levels of toxicity following PDT with **HCurc I** and **HCurc II**. However, the low toxicity observed for the Ru(II)-arene derivatives supports the possible use of these molecules for PDT.

### 2.5. Antimicrobial Activity of Curcumin and Curcumin Derivatives

Antimicrobial PDT (aPDT) is considered a promising approach to counteract the selection and spreading of multidrug-resistant bacterial strains [66]. Among natural antimicrobial photosensitizers, **CUR** was mostly investigated in Gram-positive strains such as *Streptococcus mutans*, *Staphylococcus aureus* (methicillin-sensitive and resistant strains), and *Enterococcus faecalis*. Among Gram-negative bacteria, few studies were performed on *Escherichia coli* and *Pseudomonas aeruginosa* [37].

In this study, the potential antimicrobial activity of **CUR** derivatives was tested in *Bacillus subtilis* ATCC 6633 and *Escherichia coli* MG1655, chosen, respectively, as Gram-positive and Gram-negative model microorganisms. In *E. coli* **CUR**, **HCurc I**, **HCurc II,** and their Ru(II)-arene derivatives did not show any intrinsic (i.e., in the dark) activity even at the highest tested concentration (100 μM). In addition, the irradiation with light at 410 nm and fluence values of 10 and 20 J/cm^2^ did not cause any killing effect. A certain degree of photoactivity was observed only upon treatment with **4**; PDT-MIC values of 100 ± 0.00 and 66.67 ± 23.57 μM were obtained upon irradiation at 10 and 20 J/cm^2^, respectively. Our results are in accordance with those in the literature, reporting the low sensitivity of Gram-negative bacteria to photoactivity by **CUR** [67,68]. Indeed, it is known that Gram-negative bacteria are usually tolerant to most PSs commonly used in aPDT and that treatment with EDTA enhances their susceptibility [69]. This could be ascribed to the different cell-wall composition: the thick peptidoglycan layer of Gram-positive bacteria seems to favor the crossing of both neutral and charged PSs, while the outer membrane in Gram-negative bacteria makes easier the entry of cationic compounds [42,70,71].

As expected, *B. subtilis* was shown to be more sensitive than *E. coli*. In dark conditions, compounds **4**, **5,** and **6** showed intrinsic toxic effects, especially compound **6**. Upon irradiation, **CUR** displayed good antimicrobial activity with PDT-MIC values ~21 and 15 μM under 10 and 20 J/cm^2^, respectively. Interestingly, photoactivated **HCurc I** and **HCurc II** were more efficient than **CUR**. Therefore, the chemical modifications of **CUR,** which led to the more lipophilic **HCurc I** and **HCurc II**, greatly influenced the activity of **CUR**, making the two curcuminoids more active. However, the addition of Ru(II)-arene complexes to **HCurc I** and **HCurc II** resulted in decreased lipophilicity and caused a decrease in their potency (Table 5). In general, for most of the tested compounds, the antimicrobial activity was light and dose-dependent (Table 5). A preliminary test showed that **HCurc I** could be activated at a low fluence of 3 J/cm^2^, showing a PDT-MIC of 25 μM, thus representing a promising molecule for aPDT.

To evaluate if the neutral character of **CUR** and **CUR, HCurc I,** and **HCurc II** and their Ru(II)-arene derivatives could selectively influence their interaction with bacterial models, a binding assay was performed.

**Figure 10:**
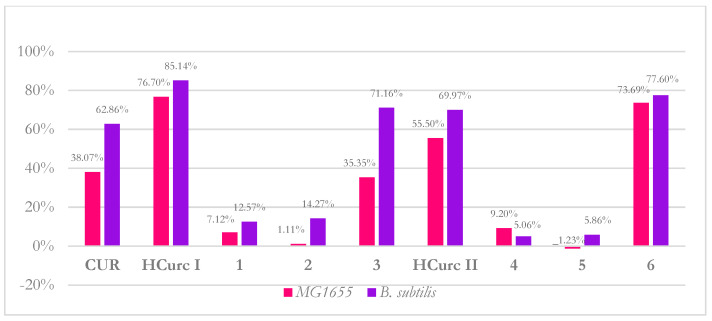
Binding rates of **CUR**, **HCurc I**, and **HCurc II** and their Ru(II)-arene derivatives in *E. coli* MG1655 and *B. subtilis* ATCC 6633.

**Figure 11:**
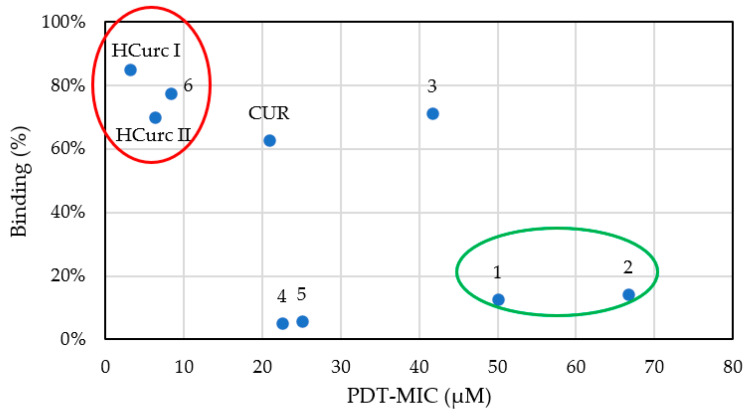
Correlation between PDT-MIC values and binding rates of **CUR**, **HCurc I**, **HCurc II**, and their Ru(II)-arene derivatives in *B. subtilis* ATCC 6633 (red circle: correlation between the highest aPDT and highest binding activity; green circle: correlation between the lowest photo-microbial and lowest binding activity).

As shown in Figure 10, derivatives **1**, **2**, **4,** and **5** were less prone to binding in both strains than the other compounds (binding rate < 15%), irrespective of bacterial cell wall anatomy. The remaining curcuminoids bind both Gram-positive and Gram-negative bacteria at a rate higher than 35%. The binding rate does not support the selective photoactivity of **CUR** and derivatives. However, in *B. subtilis,* a correlation between PDT-MIC values and binding was observed (Figure 11). It is noteworthy that the highest photo-antimicrobial activity was observed for the compounds that showed the highest binding rate (red circle), while the lowest activity was shown for compounds characterized by the lowest binding rate (green circle), respectively. Even if miniscule differences were observed at the binding level, *B. subtilis* was more sensitive than *E. coli*, according to the extended literature reporting that Gram-positive bacteria are usually more sensitive to photoinactivation by neutral compounds than Gram-negative ones [72,73]. Although the binding rate seems not to be related to the bacterial selectivity of PSs, it could help researchers choose the ideal PS for sensitive ones, such as Gram-positive bacteria.

## 3. Materials and Methods

### 3.1. Reagents and Chemicals

All the reagents and chemicals, unless otherwise indicated, were purchased by Euroclone (Milan, Italy).

### 3.2. Ruthenium(II)-Arene Derivatives Characterization

Details of the synthesis of two curcuminoids (**HCurc I** and **HCurc II**) and their Ru(II)-arene derivatives (Figure 1) can be found elsewhere [34].

#### 3.2.1. Lipophilicity Evaluation

The lipophilicity of **CUR** derivatives was estimated using a validated chromatography-based method through the use of an Agilent 1100 Series HPLC System (Santa Clara, CA, USA) equipped with an Ascentis Express C18, 5 μm HPLC column measuring 25 cm × 4.6 mm (Supelco, Bellefonte, PA, USA). The detection of **CUR** derivatives was performed using a UV-Vis spectrometer at 400 nm. For the analysis, 20 μL of DMSO solutions (1 mM) of each compound were injected into the HPLC apparatus, and retention times were evaluated. Furthermore, standard molecules with known LogP were used as references: *p*-Anisaldehyde (Sigma-Aldrich, Milano, Italy, LogP = 1.76, Abs_270nm_), Anthracene (Fluka, Milan, Italy LogP = 4.56, Abs_345nm_), 9-Anthraldehyde (Fluka, LogP = 3.347, Abs_400nm_), and Benzaldehyde (Fluka, LogP = 1.64, Abs_270nm_). A standard curve, correlating the retention time to the obtained LogP of each standard, was then drawn and used to estimate **CUR** derivatives’ LogP.

#### 3.2.2. Photobleaching Assay

The rate of absorbance decay for each derivative, due to white light exposure, was determined through the photobleaching assay. Compounds were diluted in PBS to obtain solutions with a final concentration of 100 μM, and irradiated using a tungsten halogen light (500 W, irradiance 22 mW/cm^2^) up to 2 h; every 20 min, aliquots were collected from each sample and analyzed spectrophotometrically at the Soret Band. The photodegradation percentage was calculated at each time point as the ratio of absorption intensity to absorption measured at t0.

#### 3.2.3. Singlet Oxygen Generation

The amount of singlet oxygen (^1^O_2_) generation for each **CUR** derivative was evaluated through an indirect assay in which 1,3-Diphenylisobenzofuran (DPBF) was used as ^1^O_2_ quencher and both green and blue LED light (12 led at 1 W, total amount of 12 W, RJ45 connection, constant current of 350 mA, irradiance of 1.9 mW/cm^2^ for green LED and 9.24 mW/cm^2^ for blue LED, respectively) were tested. Briefly, 50 μM DPBF and 5 μM **CUR** derivative solutions were prepared and analyzed as previously reported [74]. The decrease in DPBF absorbance at 410 nm was observed in a Varian Cary 50 Scan UV Visible Spectrophotometer (each 20 s for 20 min). The obtained absorption spectra were used to calculate ^1^O_2_ using the following equation and the singlet oxygen production of **CUR** as a reference:

^1^O_2_ = ^1^O_2_^ref^ × k/k_ref_ × f^ref^/f

The rate constant k was determined assuming pseudo-first-order kinetics and represented the slope of the DPBF degradation curve; f is the absorption factor [75].

### 3.3. Studies in Cancer Cell Lines

#### 3.3.1. Cancer Cell Lines and In Vitro Culture Conditions

HCT116 and HT29 cells were originally obtained from ATCC (American Type Culture Collection, Manassas, VA, USA) and maintained under standard culture conditions (37 °C; 5% CO_2_) in DMEM medium, supplemented with 10% fetal calf serum, 1% glutamine and 1% antibiotic mixture, 1% sodium pyruvate, and 1% non-essential amino acids. The HCT116 E6 cell line was originally obtained by transfecting HCT116 cells with the pCMVneo-E6 plasmid containing the HPV16-E6 human gene (kindly provided by Dr. B. Vogelstein, Johns Hopkins University, Baltimore, MD, USA) and was maintained in ISCOVE medium supplemented with 10% FBS and geneticin (500 μg/mL).

#### 3.3.2. Effects on Cell Viability

The antiproliferative effects of **HCurc I**, **HCurc II,** and their Ru(II)-arene derivatives were assessed using the MTT assay, as previously reported [55]. Following 24 h treatment with increasing concentrations of the compounds, cells were photoactivated by irradiation under visible light or blue LED light for 30 min and incubated in drug-free medium for 24 h in the dark. Then, 0.4 mg/mL final concentration of MTT solution was added to each well for 3 h at 37 °C, formazan crystals were dissolved in DMSO, and optical densities were measured at 570 nm using an iMark Microplate Reader (Bio-RAD, Segrate (MI), Italy). Possible non-photodynamic effects on cell growth/viability of the compounds were assessed by omitting the irradiation step.

IC_50_ values (i.e., PS concentrations reducing the cell viability by 50%) were obtained by nonlinear regression analysis using GraphPad PRISM 4.03 software (GraphPad Software Inc., San Diego, CA, USA).

#### 3.3.3. Flow Cytometric Analysis

The induction of apoptotic and necrotic cell death by the studied compounds was evaluated following 24 h exposure to the drugs at concentrations corresponding to their respective IC_50_ values, 30 min irradiation in drug-free PBS, and 24 h incubation in drug-free medium. To assess the percentage of necrotic cells at the end of treatment, cells were harvested, washed in PBS, and DNA was stained with a solution of propidium iodide (PI) in PBS (50 µg/mL) in the presence of RNAse A (30 U/mL) at room temperature for 15 min before analyzing the samples. To determine the percentage of apoptosis, cells were fixed in 70% ethanol at −20 °C for at least 20 min before staining with PI.

Intracellular ROS levels were evaluated using 2,7-dichlorodihydrofluoresceindiacetate (DCFH-DA) as a probe. At the end of the irradiation period, cells were detached, washed, and incubated in DCFH-DA (10 μM in PBS) for 45 min in the dark at 37 °C; samples were then analyzed.

A FACSCalibur flow cytometer (Becton Dickinson, Mountain View, CA, USA) was used for the analysis, and the data were processed using CellQuestPro 3.0 software (Becton Dickinson). Fluorescent emission of PI was collected through a 575 nm band-pass filter, and the percentage of apoptotic cells in each sample was determined based on the sub-G1 peaks detected in monoparametric histograms acquired in log mode. Fluorescein fluorescence was collected through a 530 nm band-pass filter, and intracellular ROS generation was quantitated in arbitrary units based on the mean fluorescence intensity (MFI).

#### 3.3.4. Assessment of Autophagy

Induction of autophagy by **HCurc I**, **HCurc II,** and their Ru(II)-arene derivatives was assessed based on the evaluation of the autophagosomal marker LC3-II protein levels by Western blot analysis of total cell extracts. Briefly, following 24 h drug treatment with PSs at concentrations corresponding to their respective IC_50_ values, 30 min irradiation, and 24 h incubation in drug-free medium, the cells were lysed, protein concentration was determined by the BCA assay, and 30 µg of protein per sample were loaded onto polyacrylamide gels (13%) and separated under denaturing conditions. Protein bands were then transferred onto Hybond-P membranes (MilliporeSigma, Milan, Italy), and Western blot analysis was performed. Equal loading of the samples was verified by re-probing the blots with a mouse monoclonal anti-β-tubulin antibody (Santa Cruz Biotechnology, Inc., Heidelberg, Germany). The immunoreactive bands were visualized by G-box (Syngene, Bengaluru, India, Chemi-XT4) using peroxidase-conjugated anti-rabbit (for LC-3II) or anti-mouse (for tubulin) secondary antibodies (Sigma-Aldrich) and the Westar Supernova Substrate (Cyanagen, Bologna, Italy).

### 3.4. Toxicity Test on G. mellonella Larvae

Gm larvae were kept in a climate chamber at a constant temperature (37 °C) and relative humidity (45 ± 10%). To assess the phototoxicity of **HCurc I**, **HCurc II** and their Ru(II)-arene derivatives, 10 µL of each compound solution was microinjected into the abdominal spiracle of the larvae with a Drummond Nanoject microinjector (Drummond Sci. Co., Broomall, PA, USA). Concentrations delivered to the larvae corresponded to the mean of the IC_50_ obtained on cancer cell lines tested and reported in Table S1. Stock solutions in DMSO of **HCurc I**, **1**, **2**, and **3**; and **HCurc II**, **4**, **5,** and **6** were diluted with sterile PBS and injected into the larvae. Once injected, larvae were incubated for 60 min in the dark, then irradiated with white light (tungsten halogen lamp, 500 W, irradiance 22 mW/cm^2^) for 2 h and once again kept in the dark at 37 °C. Survival rates were recorded every 24 h, for one week after PDT treatment. Possible intrinsic toxicity of the studied compounds was assessed in larvae treated with the same concentrations, but by omitting the irradiation step.

In addition, as a further control test, larvae were injected with 10 µL of DMSO solution in PBS (500 µM final concentration) or sterile PBS and then exposed to light or kept in the dark under the same conditions. Morphologic changes related to body darkening and the mortality of larvae were evaluated. Mortality was defined as a complete loss of mobility, verified by physical stimulation with tweezers. For all molecules, averages ± SD were obtained from four independent assays with five healthy larvae each.

### 3.5. Studies in Microorganisms

#### 3.5.1. Microorganisms and Culture Conditions

*Escherichia coli* MG1655 and *Bacillus subtilis* ATCC 6633 were chosen as Gram-negative and Gram-positive models, respectively. Both strains were grown in Luria-Bertani broth in liquid or solid form (1.5% agar) at 37 °C.

#### 3.5.2. Microbiological Assays

The photo-spot test previously optimized by our group [76] was changed accordingly to investigate the potential photoactivity of **CUR**, **HCurc I**, **HCurc II,** and their Ru(II)-arene derivatives. In a 96-well microplate, each compound was two-fold diluted (from 100 μM up to 1.5 μM) in 100 μL of LB medium. A volume of 100 μL of bacterial sample was added to each well to reach the final bacterial concentration of ~10^5^ cfu/mL. Untreated and DMSO-treated cells were included as controls. To allow the interaction between the studied compounds and microbial cells, the microplates were incubated for 30 min in the dark. Thereafter, cells were inoculated by replica-plating on LB agar and incubated in the dark or irradiated under light at 410 nm (100 mW) at increasing fluence rates (10 and 20 J/cm^2^). The cells were then incubated overnight at 37 °C. To evaluate the intrinsic toxicity of **CUR** and **CUR** derivatives, the growth spots of treated cells and cells incubated in the dark were checked and compared to those of untreated controls and controls incubated in the dark. The lowest concentration that inhibited bacterial growth was considered the MIC (minimal inhibitory concentration). To evaluate their potential photoactivity under different fluence values, the corresponding lowest concentration that inhibited the bacterial growth was considered as PDT-MIC (photodynamic treatment-minimal inhibitory concentration). The experiments were performed at least in triplicate.

#### 3.5.3. Binding Assay

The amount of **CUR** and **CUR** derivatives bound to microbial cells was evaluated by spectrophotometric analysis. Briefly, bacteria were grown overnight in LB medium at 37 °C; 1 mL volume was centrifuged at 5000 rpm for 5 min. The pellet was then resuspended in 1 mL of phosphate buffer (KH_2_PO_4_/K_2_HPO_4_ 10 mM, pH 7.4) and 10-fold diluted in phosphate buffer. The compounds were added at a final concentration of 25 μM, and the suspensions were incubated at room temperature for 1 h in the dark. Upon incubation, cells were centrifuged (6000 rpm for 10 min), and the supernatants were spectrophotometrically analyzed. A second wash was performed (6000 rpm for 10 min), and the supernatants were again spectrophotometrically analyzed. A calibration curve (μM vs. OD) was obtained for **CUR** and **CUR** derivatives. The amount of compounds not bound to bacterial cells was inferred by interpolating the data on the calibration plot. The binding rate of each compound was obtained and expressed as a percentage for *E. coli* and *B. subtilis*, respectively.

### 3.6. Statistical Analysis

Statistical analysis of all biological data was performed by means of one- or two-way ANOVA, with Bonferroni’s test for multiple comparisons, using GraphPad PRISM 4.03 software.

## 4. Conclusions

Among others, curcumin (**CUR**) is known for its application in the photodynamic field [77], but its poor stability and low bioavailability limit its use. Thus, in this work, to increase its bioavailability, **CUR** derivatives are evaluated. In particular, two molecules belonging to the curcuminoid family, **HCurc I** and **HCurc II**, in which the two hydroxyl groups of **CUR** are replaced by hydrogen atoms (**HCurc II**) or methoxy groups (**HCurc I**), are studied. Also, since data in the literature show that an increase in **CUR**/curcuminoid stability can be obtained through the coordination of the diketone system with a metal [78], these modified curcuminoids were also used as ligands to synthesize a series of Ru(II)-arene complexes. Additionally, metal-based PSs, especially Ru(II) complexes, have recently attracted considerable attention, as shown by the use of Ru(II)-PSs TLD-1433 in phase II clinical trials for bladder cancer [23,24]. Thus, in this work, for each of the two curcuminoids, a series of three aromatic compounds was coordinated with Ru(II) (cymene, benzene, and hexamethylbenzene, Figure 1) to form six metal complexes that were evaluated in a series of experiments.

Considering the antitumoral effects, in agreement with our earlier study [34], after replacing the **CUR** hydroxyl groups with methoxy groups, the obtained compound **HCurc I** and its Ru(II)-arene complexes have increased antitumor activity compared to **HCurc II** and its related complexes. For **HCurc I** and **HCurc II,** this is also true when considering the photodynamic effects of the compounds; however, similar or better photodynamic activity was observed for the six metal complexes, **1**, **2**, **3**, **4**, **5,** and **6**, compared to their precursors (namely **HCurc I** and **HCurc II**). Furthermore, their photodynamic activity was also independent of the different arene substituents. Interestingly, the six Ru(II)-arene derivatives did not show significant in vivo toxicity on the alternative animal model *Galleria mellonella*. Overall, the results reported indicate that the Ru(II)-arene derivatives are worthy of further investigation and that these compounds could represent an interesting alternative for cancer PDT.

In this study, the potential antimicrobial activity of the **CUR** derivatives was also tested in *Bacillus subtilis* ATCC 6633 and *Escherichia coli* MG1655, to represent, respectively, Gram-positive and Gram-negative model microorganisms. From these experiments, the photoantimicrobial activity of **HCurc I** against Gram-positive bacteria was indeed promising and deserves deeper investigation.

## Figures and Tables

**Table 1:** Extrapolated LogP values and ^1^O_2_ generation for **CUR; HCurc I**, **1**, **2**, and **3**; and **HCurc II**, **4**, **5,** and **6** (* data normalized on Rose Bengal).

Compound	LogP	^1^O_2_ *
**CUR**	2.67	0.19
**HCurc I**	3.55	0.02
**1**	2.74	0.08
**2**	1.6	0.14
**3**	2.85	0.03
**HCurc II**	4.48	0.01
**4**	3.21	0.08
**5**	2.11	0.11
**6**	3.39	0.14

**Table 2:** Photodegradation of **CUR**; **HCurc I**, **1**, **2**, and **3;** and **HCurc II**, **4**, **5,** and **6** following irradiation with a white halogen lamp.

Time (min)	CUR	HCurc I	1	2	3	HCurc II	4	5	6
10	100%	57.1%	100%	100%	100%	31.2%	100%	100%	100%
20	100%	25.2%	100%	100%	100%	14.7%	100%	100%	100%
30	73.6%	0%	76.2%	79.7%	81.1%	0%	66.7%	62.9%	65.3%
60	60.9%	0%	68.6%	67.5%	80.1%	0%	59.9%	57.9%	59.6%
90	55.4%	0%	63.2%	62.1%	76.9%	0%	52.6%	57.5%	47.3%
120	49.6%	0%	61.9%	61.1%	71.8%	0%	50%	57.5%	44.6%

**Table 3:** IC_50_ values obtained in HCT116 and HT29 cell lines following 24 h treatment with **CUR**, **HCurc I,** and **HCurc II**, 30 min irradiation (light) or not (dark) with a halogen white light lamp, 24 h incubation in drug-free medium, and MTT assay (mean ± E.S. of 3/5 independent experiments: * *p* < 0.05 vs. **CUR**).

IC_50_ (μM)	HCT116		HT29	
	Dark	Light	Dark	Light
**CUR**	7.43 ± 1.24	1.05 ± 0.19	8.43 ± 1.31	3.83 ± 0.44
**HCurc I**	3.01 ± 0.28	1.06 ± 0.23	14.57 ± 1.09	1.59 ± 0.27 *
**HCurc II**	49.14 ± 1.18	1.91 ± 0.14	25.95 ± 2.55	3.36 ± 0.74

**Table 4:** Phototoxic index of **HCurc I**, **HCurc II,** and their Ru(II)-arene derivatives in HCT116 and HT29 cell lines.

	HCT116	HT29
**HCurc I**	2.8	9.1
**1**	4.6	5.5
**2**	2.0	12.5
**3**	3.6	8.6
**HCurc II**	23.5	7.7
**4**	4.1	5.4
**5**	3.1	7.1
**6**	2.8	4.7

**Table 5:** Effect of **CUR, HCurc I,** and **HCurc II** and their Ru(II)-arene derivatives on *Bacillus subtilis*. The compounds were administered at decreasing concentrations (from 100 to 1.5 μM) to cells at 10^5^ cfu/mL. Cells were incubated in the dark for 30 min and inoculated on LB agar. After dark incubation or irradiation under light at 410 nm (10 or 20 J/cm^2^), bacteria were grown at 37 °C for 24 h, and growth spots were checked. MIC and PDT-MIC values indicate the lowest concentrations that compromised bacterial growth under dark conditions or irradiation, respectively (means ± SD of at least three independent experiments).

	0 J/cm^2^	10 J/cm^2^	20 J/cm^2^
**CUR**	>100	20.83 ± 5.89	14.58 ± 7.80
**HCurc I**	>100	3.13 ± 0.00	3.13 ± 0.00
**1**	>100	50 ± 0.00	31.25 ± 10.83
**2**	>100	66.67 ± 23.57	50 ± 0.00
**3**	>100	41.67 ± 11.79	29.17 ± 6.25
**HCurc II**	>100	6.25 ± 4.42	2.60 ± 0.78
**4**	50 ± 0.00	22.50 ± 5.00	13.75 ± 3.13
**5**	100 ± 0.00	25 ± 0.00	22.5 ± 6.25
**6**	12.5 ± 0.00	8.33 ± 2.95	6.25 ± 0.00

## Data Availability

Data will be made available on request.

## Supplementary Materials

The following supporting information can be downloaded at: https://www.mdpi.com/article/10.3390/molecules28227537/s1, Table S1: IC_50_ values obtined in HCT116 and HT29 cell lines following 24 h treatment with **CUR**, **HCurc I** and **HCurc II**, 30 min irradiation with halogen white light lamp, 24 h incubation in drug-free medium and MTT assay (mean ± E.S. of 3/5 independent experiments); Figure S1: IC_50_ values obtained in HCT116 cells following 24 h treatment with the studied compounds, 30 min irradiation under blue LED or white halogen lamp, 24 h incubation in drug-free medium and MTT assay (mean ± ES 3/4 independent experiments; *** *p* < 0.001 vs. HCurc@ same condition; # *p* < 0.01 vs. HCurc@ same condition and dark; ** *p* < 0.01 vs HCurc I and 2 same condition; § *p* < 0.001 vs dark); Table S2: Phototoxic index of **HCurc I**, **HCurc II** and their arene-Ru(II) derivatives in HCT116 E6 cells; Table S3: Effects of **HCurc I**, **1**, **2** and **3** on Gm survival. d: days after treatment; C: control (PBS injection); D subscript: dark condition; L subscript: after PDT. (means ±SD of four independent assays); Table S4: Effects of **HCurc II**, **4**, **5** and **6** on Gm survival. d: days after treatment; C: control (PBS injection); D subscript: dark condition; L subscript: after PDT. (means ±SD of four independent assays).
