# Supplementary material for: Ruthenium(II)-Arene Curcuminoid Complexes as Photosensitizer Agents for Antineoplastic and Antimicrobial Photodynamic Therapy: In Vitro and In Vivo Insights
Source: Molecules. 2023 Nov 11;28(22):7537. doi: 10.3390/molecules28227537 (PMC10673066; doi:10.3390/molecules28227537)
Supplement: Supplementary file 1 [file molecules-28-07537-s001.zip › molecules-2697148-supplementary.pdf]

Supplementary Materials

Table S1. IC_{50} values obtined in HCT116 and HT29 cell lines following 24h treatment with **CUR**, **HCurc I** and **HCurc II**, 30 min irradiation with halogen white light lamp, 24h incubation in drug-free medium and MTT assay (mean ± E.S. of 3/5 independent experiments).

**IC_{50} (mM)**	**HCT116**	**HT29**
**HCurc I**	1.06±0.23	1.59±0.27
**1**	3,75±1,21	5,25±1,31
**2**	15,76±1,85	3,58±0,61
**3**	4,11±0,81	5,80±1,15
**HCurc II**	1.91±0.14	3.36±0.74
**4**	6,97±1,36	10,610±1,51
**5**	6,62±1,51	8,154±1,53
**6**	10,03±0,89	10,651±1,15

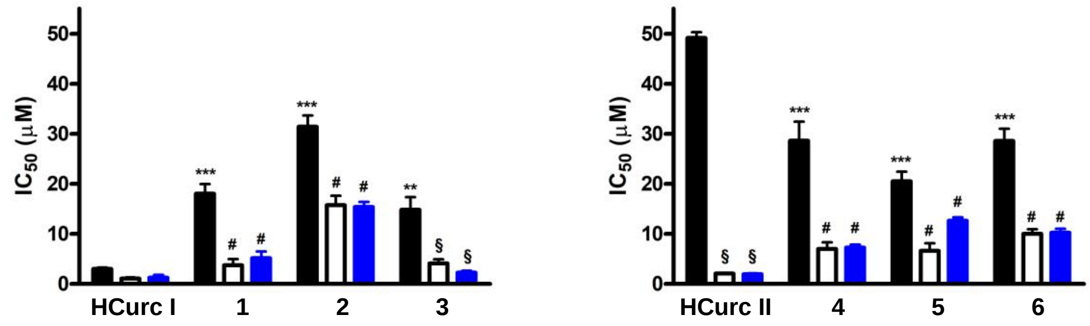

Figure S1. IC_{50} values obtained in HCT116 cells following 24 h treatment with the studied compounds, 30 min irradiation under blue LED or white halogen lamp, 24 h incubation in drug-free medium and MTT assay (mean ± ES 3/4 independent experiments; *** p<0.001 vs HCurc@ same condition; # p<0.01 vs HCurc@ same condition and dark; ** p<0.01 vs HCurc@ and 2 same condition; § p<0.001 vs dark)

Table S2. Phototoxic index of **HCurc I**, **HCurc II** and their arene-Ru(II) derivatives in HCT116 E6 cells.

**HCurc I**	**1**	**2**	**3**	**HCurc II**	**4**	**5**	**6**
6.6	10.32	31.9	8.6	46.81	5.6	19.51	4.1

Table S3. Effects of **HCurc I**, **1**, **2** and **3** on Gm survival. d: days after treatment; C: control (PBS injection); D subscript: dark condition; L subscript: after PDT. (means ±SD of four independent assays).

**HCurc I and derivatives**									
**d**	**% survival *G. mellonella* (means ± SD)**								
	C	HCurc I_D	HCurc I_L	1_D	1_L	2_D	2_L	3_D	3_L
1	100 ± 0	80 ± 0	50 ± 11.5	100 ± 0	100 ± 0	100 ± 0	95 ± 10	100 ± 0	100 ± 0
3	100 ± 0	80 ± 0	45 ± 10	95 ± 10	100 ± 0	90 ± 20	95 ± 10	95 ± 10	100 ± 0
5	100 ± 0	80 ± 0	40 ± 0	95 ± 10	100 ± 0	90 ± 20	95 ± 10	95 ± 10	100 ± 0
7	100 ± 0	75 ± 10	25 ± 10	95 ± 10	100 ± 0	90 ± 20	95 ± 10	95 ± 10	100 ± 0

Table S4. Effects of **HCurc II**, **4**, **5** and **6** on Gm survival. d: days after treatment; C: control (PBS injection); D subscript: dark condition; L subscript: after PDT. (means ±SD of four independent assays).

**HCurc II and derivatives**									
**d**	**% survival *G. mellonella* (means ± SD)**								
	C	$HCurc\ II_D$	$HCurc\ II_L$	4_D	4_L	5_D	5_L	6_D	6_L
1	100 ± 0	95 ± 10	75 ± 10	100 ± 0	95 ± 10	100 ± 0	100 ± 0	95 ± 10	100 ± 0
3	100 ± 0	85 ± 10	45 ± 10	100 ± 0	90 ± 11.5	100 ± 0	100 ± 0	95 ± 10	100 ± 0
5	100 ± 0	80 ± 0	30 ± 11.5	100 ± 0	85 ± 19.1	100 ± 0	100 ± 0	95 ± 10	100 ± 0
7	100 ± 0	75 ± 10	0	100 ± 0	85 ± 19.1	100 ± 0	100 ± 0	95 ± 10	100 ± 0
